# Osteoporosis Knowledge and Awareness Among Patients and Accompanying Attendants: A Cross-Sectional Study in a Physical Medicine and Rehabilitation Department

**DOI:** 10.7759/cureus.75901

**Published:** 2024-12-17

**Authors:** Arvind K Sharma, Satyasheel S Asthana, Ivanah P Nongrum

**Affiliations:** 1 Physical Medicine and Rehabilitation, All India Institute of Medical Sciences, Raebareli, Raebareli, IND

**Keywords:** bone mineral density (bmd), fall prevention, fracture, musculoskeletal rehabilitation, neurorehabilitation, osteoporosis, osteoporosis knowledge and awareness, physical medicine and rehabilitation (pm&r), spinal cord injury (sci), stroke

## Abstract

Introduction: Osteoporosis is a common yet underdiagnosed condition that increases fracture risk and disability. It is particularly prevalent in individuals with musculoskeletal and neurological disorders due to factors like immobility and disuse. Despite its impact, awareness of osteoporosis remains low, especially in this high-risk population. This study aims to assess the knowledge of osteoporosis among patients with neurological and musculoskeletal disorders attending a Physical Medicine and Rehabilitation outpatient department (OPD). Using tools like the Osteoporosis Knowledge Assessment Tool- Hindi (OKAT-H), the study seeks to identify knowledge gaps and promote prevention and early detection.

Materials and methods: A cross-sectional study was conducted, involving 300 participants, including patients and accompanying attendants, who visited the OPD of Physical Medicine and Rehabilitation, aged 40 years and above. The sample size was calculated based on data from a previous study, resulting in a target of 292 participants. Socioeconomic status was assessed using the Modified Kuppuswamy scale, classifying participants into five classes. Osteoporosis awareness was measured using the OKAT-H.

Results: The mean OKAT-H score was found to be 9.82 out of 20 (49.1%), with a standard deviation of 5.06, ranging from a minimum score of 2/20 to a maximum of 20/20. Significant differences in OKAT-H scores were found in rural and urban populations (P value=0.001) and among the classes of socioeconomic status (P value=0.001) of the participants based on the Modified Kuppuswamy scale.

Conclusion: Our study using the OKAT-H scale revealed a significant knowledge gap regarding osteoporosis among individuals with neurological and musculoskeletal disorders, as well as their caregivers. This lack of awareness increases the risk of fractures and related complications. To address this, targeted educational programs should be implemented in the OPD of Physical Medicine and Rehabilitation departments, focusing on improving knowledge of osteoporosis, raising awareness of its risks and prevention, and educating individuals on fall prevention measures, the use of mobility and orthotic aids, and necessary home and workplace modifications.

## Introduction

Osteoporosis is defined as diminished bone mass per unit volume, resulting in increased fragility. The World Health Organization has defined osteoporosis as bone mineral density (BMD) of 2.5 standard deviations below the peak mean bone mass of young, healthy adults [1]. Osteoporosis is a silent condition contributing to decreased bone mass and deterioration of the structure of bones, thereby making the individual more susceptible to fractures due to higher fragility of bones. It has been reported that 61 million people in India have osteoporosis, out of which 80% are women [2]. Osteoporosis is associated with an increased rate of disability, health care costs, morbidity and mortality [3]. Globally, osteoporosis has been reported to cause about nine million fractures per year [4]. Osteoporosis could also lead to musculoskeletal complaints such as back pain and leg pain, thus having a negative effect on quality of life [5]. Patients presenting with these symptoms for musculoskeletal rehabilitation in the Department of Physical Medicine and Rehabilitation (PMR) may have compromised bone health.

Risk factors associated with osteoporosis include increasing age, previous history of steroid medication, consumption of large amounts of alcohol or caffeine, other family members having history of fractures due to osteoporosis, immobilization, sedentary lifestyle, and low calcium and vitamin D intake [6]. Thus, knowledge and understanding of the risk for osteoporosis have been shown to help in the prevention of osteoporosis [7].

The patients attending the outpatient facility (OPD) of the PMR Department primarily present with musculoskeletal complaints like low back pain, knee and shoulder pain, polyarthralgia, non-specific generalized body pain, etc. Some of the most common presentations seen in PMR OPD include osteoarthritis, rheumatoid arthritis, myofascial pain syndrome, fibromyalgia and axial spondyloarthropathy. Rehabilitation, using health-based approaches has been seen to facilitate optimization of physical function and encourage participation in persons with physical impairments and limitations in patients of spinal cord injury (SCI), cerebrovascular accident (CVA), traumatic brain injury (TBI), and Guillain-Barré syndrome (GBS) and musculoskeletal conditions (including low back pain and neck pain) [8]. These disorders may be responsible for activity limitations and participation restrictions leading to sedentary life and immobilization. SCI patients are mostly bedridden or wheelchair-bound and loss of mechanical loading is a major contributory cause for development of osteoporosis. SCI is featured by disuse, causing loss of biomechanical stress on bones leading to an adaptive response where there is inhibition of osteoblastic bone formation and increases in osteoclastic bone resorption, thus resulting in demineralization [9]. The rate of fractures in the SCI population is double that of the general population [9]. 

Osteopenia and osteoporosis are more prevalent in young adults with acquired brain injury (ABI) than in the general population [10]. Patients who were able to walk outdoors exhibited significantly higher BMD at the femoral neck compared to those who could only walk indoors or those who were unable to walk (p < 0.001) [10]. Osteoporosis is also more common in patients with neurodegenerative conditions such as Alzheimer’s disease, Parkinsonism, amyotrophic lateral sclerosis and multiple sclerosis [11]. The use of medications commonly prescribed to these patients, including antidepressants, sedatives, antihypertensives, and the practice of polypharmacy (the concurrent use of multiple medications), can also elevate the risk of falls. [12]. Older age is linked to decreased balance, reduced mobility, and impairments in vision and cognition, all of which contribute to an increased risk of falls. [12]. Globally, a third of people aged 65 years and older fall at least once a year, with 5% of these falls resulting in fractures [12]. With advancements in medical care, individuals with intellectual and developmental disabilities are living longer and are increasingly facing age-related conditions, such as osteoporosis, that typically affect the elderly population [13].

Osteopenia and osteoporosis are highly prevalent among adults with disabilities undergoing rehabilitation, in comparison to the general young adult population. The duration of disability and mobility status are independent factors influencing BMD at the hip. Therefore, bone health monitoring should be an integral part of the long-term care for adults with newly acquired disabilities [14].

Based on the above literature review, it becomes increasingly imperative to assess the current status of knowledge and awareness about osteoporosis in this cohort of patients who are already disabled or vulnerable to developing some form of disability in the future. Understanding their level of awareness is crucial, as osteoporosis may exacerbate existing disabilities or increase the risk of future complications. Gaining insight into their knowledge helps identify gaps in education and awareness, allowing for the development of targeted interventions that can prevent or manage osteoporosis more effectively, thereby improving overall health outcomes and quality of life for this vulnerable population.

On reviewing available literature, it was found that despite osteoporosis being a major health concern, the amount of understanding regarding this condition among the public is very low [15]. In a previous survey conducted by the International Osteoporosis Foundation (2000), it was found that four out of five women suffering from osteoporosis were unaware of their risk before diagnosis [7]. In the study by Kadam et al., it was observed that no significant difference was observed between the male and female participants in Osteoporosis Knowledge Test scores [7]. In a study on awareness of osteoporosis in the male population by Chaudhary et al., it was found that 42.14% of males were aware of osteoporotic fractures [16]. While numerous studies have evaluated awareness levels among postmenopausal women, as well as in the general population (including both male and female participants) and of male participants alone, there is a notable lack of research focused on the awareness levels in the specific cohort of patients visiting the Physical Medicine and Rehabilitation OPD for neurorehabilitation and musculoskeletal rehabilitation. This cohort consists of individuals with various neurological and musculoskeletal disorders that may cause activity limitation and participation restriction. These conditions often involve complex interactions between physical, cognitive, personal, and environmental factors which could influence individuals' awareness of their health and limitations.

Rehabilitation is often viewed as a cyclical process that begins with identifying the patient’s issues and needs, followed by understanding how these problems relate to personal and environmental factors. This is followed by setting therapy goals, planning and executing suitable interventions, and evaluating their outcomes [17]. The World Report on Disability defines rehabilitation as a series of actions aimed at helping individuals who have or are at risk of developing a disability to attain and sustain the highest possible level of functioning in relation to their environment [18].

Therefore, to prevent disability associated with osteoporosis, our study aims to fill this gap by assessing the level of awareness in this particular population. The initial action to be taken should include steps to assess the existing knowledge regarding this condition, especially of those affected by it. Studies on other diseases have shown that knowledge of symptoms can facilitate early diagnosis, and awareness of risk factors can promote lifestyle and behaviour modifications [7].

There are a number of tools used to assess awareness and understanding of osteoporosis, such as the Osteoporosis Knowledge Assessment Tool (OKAT) [19], Facts on Osteoporosis Quiz (FOOQ) [20], and Osteoporosis Knowledge Test (OKT) [21].

However, use of survey questionnaires in the local vernacular language is essential for improving participation rates and obtaining an unbiased sample [22]. Hence, we have used the OKAT-Hindi (OKAT-H) questionnaire as our study tool in the language well understood by the local population attending the OPD of the Department of Physical Medicine and Rehabilitation.

## Materials and methods

Inclusion and exclusion criteria

A cross-sectional study was carried out on patients and accompanying attendants, attending the outpatient facility of the Department of Physical Medicine and Rehabilitation at All India Institute of Medical Sciences (AIIMS) Raebareli, Uttar Pradesh. Approval from the Institutional Ethics Committee (IEC), AIIMS Raebareli (F.3A/BIOETHICS/AIIMS-RBL/APPR/IMP/2024-8/11) was taken prior to enrollment of subjects in the study. Inclusion criteria were patients and accompanying attendants aged 40 years and above, willing to be included in the study. All participants gave informed written consent prior to being included in the study and filled out the self-administered questionnaire. Patients were enrolled in the study from 4th November 2024 till 3rd December 2024. 

The sample size of this study was calculated based on a previous study, where the level of knowledge regarding osteoporosis was about 37.1%, and using the formula 4pq/d2, where P is the prevalence of poor awareness, q = 1 − p, and d is the allowable error, that is, 15% of prevalence, the sample size was calculated to be 292 [23].

Assessment of socioeconomic status

The socioeconomic status of the participants was assessed using the Modified Kuppuswamy scale [24]. This is based on the following three variables: Monthly total family income, occupation of the head of the family and education of the head of the family. Based on this, participants were classified into five socioeconomic classes, namely Upper, Upper middle, Lower middle, Upper lower and Lower.

Assessment of osteoporosis awareness

Knowledge regarding osteoporosis was assessed using the OKAT-H questionnaire. OKAT-H is a useful tool to assess knowledge about osteoporosis. It consists of 20 questions [5]. The first 12 questions are with regard to general understanding of osteoporosis; questions 13-17 are related to contributory risk factors of osteoporosis and the last three questions assess knowledge regarding treatment options and osteoporosis prevention [19]. The 20 questions can be answered with one out of three responses: ‘True,’ ‘False’ and ‘I do not know.’ The responses of the patients were evaluated against the correct answers of the questionnaire. Every correct response was awarded one point and every wrong and unanswered question was given zero points.

We used the Hindi version of OKAT, OKAT-H, which has Cronbach’s alpha of 0.892, suggestive of high internal consistency [5].

The OKAT-H percentage scores were calculated and grouped as: <20%: Very poor, 21%-40%: Poor, 41%-60%: Average, 61%-80%: Good, and 81%-100%: Very good [23].

Statistical analysis

Data analysis was done by using SPSS 26 (IBM Corp., Armonk, NY, USA). Chi-square test was used for categorical variables. 

## Results

A total of 300 responses were recorded. Out of the 300 participants, 154 (51.3%) were men and 146 (48.7%) were women. The mean age of the participants was calculated to be 52.23 years with a standard deviation of 10.92. Based on the results compiled, no significant difference in gender distribution of participants was found (p value 0.644). There was no significant difference in socioeconomic status of the study population (p value 0.343). There was a significant difference in the diagnosis of the patients accompanied by the individuals (p value 0.0012) where it was found that 59.3% of the participants were accompanying patients with musculoskeletal (MSK) complaints including low back pain, osteoarthritis of knee and other musculoskeletal complaints. 40.7% of participants were accompanying patients with neurological disorders with significant limitation in ambulation and mobility (including SCI, CVA, TBI and cerebral palsy). Demographical variables consisting of gender, age groups, socioeconomic status from the Modified Kuppuswamy scale, diagnosis and rural versus urban population are depicted in Table 1.

**Table 1:** Demographical variables of participants including gender, age, socioeconomic status according to Modified Kuppuswamy scale, rural and urban population and diagnosis of patients they are accompanying. P value was calculated using Chi-Square test.

Demographic parameters	Variables	Number of participants	Percentage	p-value (Chi-Squared value)
Gender	Male	154	51.3	0.644 (0.2133)
	Female	146	48.7	
Socioeconomic status based on Modified Kuppuswamy scale	Lower	70	23.3	0.343 (4.500)
	Upper Lower	64	21.3	
	Lower Middle	63	21.0	
	Upper Middle	52	17.3	
	Upper	51	17.0	
Diagnosis of the patient accompanied by the participant	Musculoskeletal (MSK) disorders	178	59.3	0.0012 (10.4533)
	Neurological disorders	122	40.7	
Age group in years	Less than 50	160	53.3	0.248 (1.3333)
	Greater than or equal to 50	140	46.7	
Rural and Urban population category	Rural	178	59.3	0.0012 (10.4533)
	Urban	122	40.7	

The mean OKAT-H score was found to be 9.82 out of 20 (49.1%) with a standard deviation of 5.06, ranging from a minimum score of 2/20 to a maximum of 20/20. The analysis of OKAT-H scores based on the residence of populations in rural and urban regions was found to have a significant difference in the scores of OKAT-H using the Chi-square test, which has been depicted in Table 2.

**Table 2:** OKAT-H scores analysis based on residence of population in rural and urban regions OKAT-H: Osteoporosis Knowledge and Assessment Tool-Hindi P value was calculated using Chi-Square test.

Population	OKAT-H score categories	<20%	21%-40%	41%-60%	61%-80%	>81%	Total	P value (Chi-squared value)
Rural	Number of participants	51	55	44	23	5	178	0.001 (82.201)
	Percentage of participants	28.7%	30.9%	24.7%	35.4%	12.9%	100%	
Urban	Number of participants	10	12	23	42	35	122	
	Percentage of participants	8.2%	9.8%	18.9%	34.4%	28.7%	100%	

OKAT-H scores based on age groups of the participants, using the Chi-square test, are depicted in Table 3. There was a significant difference found using the Chi-square test in the OKAT-H scores of the various classes of socioeconomic status based on the Modified Kuppuswamy scale as depicted in Table 4. OKAT-H scores based on gender, using the Chi-square test, are depicted in Table 5. OKAT-H scores were analyzed based on diagnosis of patients accompanied using Chi-square test as depicted in Table 6.

**Table 3:** OKAT-H scores analysis based on age groups of population. OKAT-H: Osteoporosis knowledge assessment tool-Hindi P value was calculated using Chi-Square test.

Population based on age groups	OKAT-H score categories	<20%	21%-40%	41%-60%	61%-80%	>81%	Total	P value (Chi-Squared value)
41-50	Number of participants	34	38	43	31	22	168	0.879 (6.668)
	Percentage of participants	20.2%	22.6%	25.6%	18.5%	13.1%	100%	
51-60	Number of participants	12	13	10	16	7	58	
	Percentage of participants	20.7%	22.4%	17.2%	27.6%	12.1%	100%	
61-70	Number of participants	12	10	12	14	9	57	
	Percentage of participants	21.1%	17.5%	21.1%	24.6%	15.8%	100%	
71-80	Number of participants	3	6	2	4	2	17	
	Percentage of participants	17.6%	35.3%	11.8%	23.5%	11.8%	100%	
Total		61	67	67	65	40	300	

**Table 4:** OKAT-H scores analysis based on socioeconomic status of population based on Modified Kuppuswamy scale. OKAT-H: Osteoporosis knowledge assessment tool-Hindi P value was calculated using Chi-Square test.

Population based on Socioeconomic status based on Modified Kuppuswamy scale	OKAT-H score categories	<20%	21%-40%	41%-60%	61%-80%	>81%	Total	P value (Chi-Squared value)
Upper	Number of participants	0	0	0	12	39	51	0.001 (796.97)
	Percentage of participants	0%	0%	0%	23.5%	76.5%	100%	
Upper middle	Number of participants	0	0	6	45	1	52	
	Percentage of participants	0%	0%	11.5%	86.5%	1.9%	100%	
Lower middle	Number of participants	0	1	58	4	0	63	
	Percentage of participants	0%	1.6%	92.1%	6.3%	0%	100%	
Upper lower	Number of participants	5	54	1	4	0	64	
	Percentage of participants	7.8%	84.4%	1.6%	6.2%	0%	100%	
Lower	Number of participants	56	12	2	0	0	70	
	Percentage of participants	80%	17.1%	2.9%	0%	0%	100%	

**Table 5:** OKAT-H scores analysis based on gender of participants. OKAT-H: Osteoporosis knowledge assessment tool-Hindi P value was calculated using Chi-Square test.

Population based on gender	OKAT-H score categories	<20%	21%-40%	41%-60%	61%-80%	>81%	Total	P value (Chi-Squared value)
Males	Number of participants	26	31	33	40	24	154	0.161 (6.569)
	Percentage of participants	16.9%	20.1%	21.4%	26%	15.6%	100%	
Females	Number of participants	35	36	34	25	16	146	
	Percentage of participants	24%	24.7%	23.3%	17.1%	11%	100%	

**Table 6:** OKAT-H scores analysis based on diagnosis of patient. OKAT-H: Osteoporosis knowledge assessment tool-Hindi P value was calculated using Chi-Square test.

Diagnosis of patient being accompanied	OKAT-H score categories	<20%	21%-40%	41%-60%	61%-80%	>81%	Total	P value (Chi-Squared value)
Musculoskeletal disorders	Number of participants	37	36	45	33	27	178	0.223 (5.70)
	Percentage of participants	20.8%	20.2%	25.3%	18.5%	15.2%	100%	
Neurological disorders	Number of participants	24	31	22	23	11	122	
	Percentage of participants	19.7%	25.4%	18%	21.3%	10.2%	100%	

Responses to the questions from OKAT-H questionnaire

**Figure 1:**
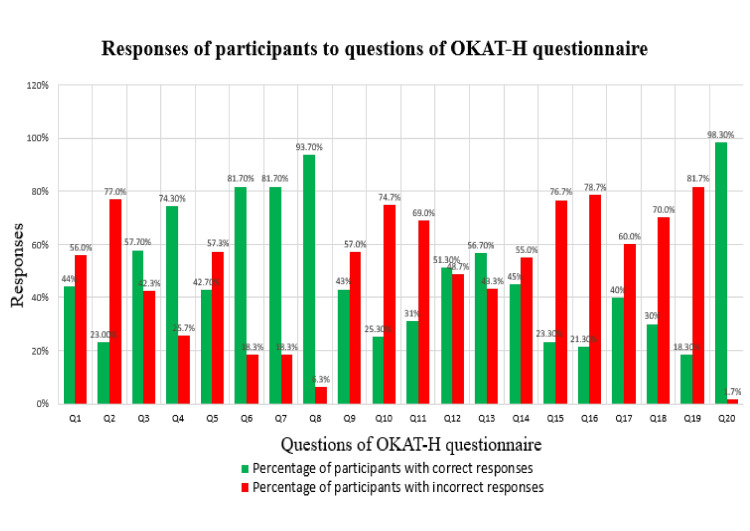
Graphical representation of the responses of the participants to the OKAT-H questionnaire with the percentage of correct and incorrect responses. OKAT-H: Osteoporosis Knowledge Assessment Tool-Hindi Q1 to Q20: Questions of OKAT-H questionnaire (listed out in Table 7)

The responses of participants to the questions of the OKAT-H questionnaire are depicted in Table 7 and a graphical representation of them is shown in Figure 1.

**Table 7:** Responses of participants to the OKAT-H questionnaire. Table contains questions of the OKAT-H questionnaire with their English translation for each of the 20 questions. OKAT-H: Osteoporosis Knowledge Assessment Tool-Hindi

Question number	Questions of OKAT-H questionnaire	Response (0=Incorrect response; 1=correct response)	Total Responses	Percentage of participants with the responses
Q1	ऑस्टियोपोरोसिस हड्डियों के कमजोर पड़ने की बिमारी है जिसमे उनकेटूटने का खतरा बढ़ जाता है I (Osteoporosis leads to an increased risk of bone fractures.)	0	168	56.0%
		1	132	44.0%
Q2	इस बिमारी में हड्डियों के टूटने के पहले दर्द जैसे लक्षण आते हैं I (Osteoporosis usually causes symptoms e.g., pain before fractures occur.)	0	231	77.0%
		1	69	23.0%
Q3	किशोरावस्था में हड्डियाँ ज्यादा मजबूत होने के बाद भी बुढ़ापे में इस बीमारी का खतरा कम नहीं होता I (Having a higher peak bone mass at the end of childhood gives no protection against the development of osteoporosis in later life.)	0	127	42.3%
		1	173	57.7%
Q4	पुरुषों में यह बिमारी ज्यादा पायी जाती है I (Osteoporosis is more common in men.)	0	77	25.7%
		1	223	74.3%
Q5	तम्बाकू सेवन से इस बीमारी का खतरा बढ़ता है I (Cigarette smoking can contribute to osteoporosis.)	0	172	57.3%
		1	128	42.7%
Q6	भारतीय महिलायों में इस रोग का खतरा बिलकुल नहीं है I (Indian women do not have any risk of developing Osteoporosis.)	0	55	18.3%
		1	245	81.7%
Q7	रोगग्रस्त हड्डियाँ मामूली से चोट से भी टूट सकती हैं I (Diseased bones can break even with minor injuries.)	0	55	18.3%
		1	245	81.7%
Q8	80 साल के उम्र तक अधिकांश महिलाएं इस रोग से पीड़ित होती हैं I (By age 80 years, a majority of women have osteoporosis.)	0	19	6.3%
		1	281	93.7%
Q9	अधिकांश महिलायों के जीवनकाल में 50 की उम्र के बाद कम से कम एक बार हड्डी टूटने की सम्भावना रहती है I (From age 50 years, most women can expect at least one fracture before they die.)	0	171	57.0%
		1	129	43.0%
Q10	किसी भी प्रकार का शारीरिक व्यायाम इस रोग की रोकथाम करता है I (Any type of physical activity is beneficial for osteoporosis.)	0	224	74.7%
		1	76	25.3%
Q11	व्यक्ति अपने जीवन शैली , स्वास्थ्य और लक्षणों के आधार पर इस रोग के होने के खतरे का अनुमान लगा सकता है I (It is easy to tell whether I am at risk of osteoporosis by my clinical risk factors.)	0	207	69.0%
		1	93	31.0%
Q12	परिवारजनों में ऑस्टियोपोरोसिस होने पर हमें इस रोग का खतरा बढ़ जाता है I (Family history of osteoporosis strongly predisposes a person to osteoporosis.)	0	146	48.7%
		1	154	51.3%
Q13	प्रतिदिन दो गिलास दूध पीने से हमें पर्याप्त मात्रा में कैल्शियम प्राप्त होता है I (An adequate calcium intake can be achieved from two glasses of milk a day.)	0	130	43.3%
		1	170	56.7%
Q14	जिन्हें दुग्ध उत्पादों से परहेज हो उनके लिए दाले , हरी पत्तेदार सब्जियां और मछलियाँ कैल्शियम के अच्छे स्रोत हैं I (Pulses, green leafy vegetables and fish are good sources of calcium for those who avoid dairy products.)	0	165	55.0%
		1	135	45.0%
Q15	केवल कैल्शियम की गोलियां ही इस रोग से बचा सकती हैं I (Calcium supplements alone can prevent bone loss.)	0	230	76.7%
		1	70	23.3%
Q16	थोड़ी मात्रा में शराब पीने से इस रोग का खतरा बढ़ता नहीं है I (Alcohol in moderation has little effect on osteoporosis.)	0	236	78.7%
		1	64	21.3%
Q17	अत्यधिक मात्रा में नमक का सेवन इस रोग का खतरा बढाता है I (A high salt intake is a risk factor for osteoporosis.)	0	180	60.0%
		1	120	40.0%
Q18	मासिक बंद होने के बाद के 10 सालो में हड्डियाँ ज्यादा कमजोर नहीं होतीँ I (There is a small amount of bone loss in the 10 years following the onset of menopause.)	0	210	70.0%
		1	90	30.0%
Q19	मासिक के बंद होने के कारण होने वाले हड्डियों की कमजोरी से हॉर्मोन थेरेपी बचाती है I (Hormone therapy prevents further bone loss at any age after menopause.)	0	245	81.7%
		1	55	18.3%
Q20	इस रोग का कारगर इलाज़ भारत में उपलब्ध नहीं है I (There are no effective treatments for osteoporosis available in India.)	0	5	1.7%
		1	295	98.3%

For general understanding of osteoporosis (first 12 questions), based on responses from the subjects compiled it was seen that only 44% (132) were aware that osteoporosis causes patients to be at a higher risk of fractures. 25.7% of the subjects correctly answered that osteoporosis is more commonly seen in women. 57.3% of the participants were unaware that cigarette smoking is a factor contributing to osteoporosis and 81.7% were aware of the association between decreased bone strength, falls, and fractures. 51.3% of the participants were aware a positive family history of osteoporosis has a positive association with chances of developing osteoporosis.

For knowledge about dietary factors and risk factors of osteoporosis (questions 13-17), 21.3% and 40% of the subjects were aware of the contributory effect of excessive alcohol consumption and high amounts of salt as risk factors for osteoporosis, respectively. 23.3% of the participants were of the opinion that calcium supplementation alone could help prevent the onset of osteoporosis.

For treatment-related knowledge (questions 18-20), 70% of participants were unaware that osteoporosis is more prevalent in postmenopausal women. Only 18.3% of the participants were aware that hormone replacement therapy (HRT) could help in limiting the progression of osteoporosis.

## Discussion

The objective of the study was to evaluate the knowledge of osteoporosis among the patients and their attendants visiting the OPD of Physical Medicine and Rehabilitation. The patients and their attendants mostly come with MSK complaints or neurological disorders and face difficulties in ambulation and are at risk for frequent falls, thereby making them more vulnerable to fractures. This is particularly seen in SCI population due to SCI-induced osteoporosis developing secondary to mechanical unloading after paralysis, resulting in inhibition of bone formation and indirect stimulation of bone resorption [25]. Such subjects were selected as they are at high risk for osteoporosis and fragility fractures, and this highlights the need for early and proactive screening in this group. In this study, the mean OKAT-H score was found to be 9.82 out of 20 (49.1%) with a standard deviation of 5.06. The participants belonging to lower socioeconomic status were found to have lesser knowledge regarding osteoporosis and its risk factors. Participating individuals demonstrated limited knowledge regarding dietary risk factors and 23.3% of the participants answered that only calcium would suffice to correct this condition and were unaware of the various methods of treating osteoporosis.

Most studies have focused on osteoporosis awareness among postmenopausal women. In Senthilraja M et al., the OKAT score was used to assess the level of knowledge among postmenopausal women in a hospital in South India [23]. It was observed that 37.1% (7.4/20) were correctly answered. In a study by Alqahtani GM et al., the OKAT score was assessed among the female population in a hospital in Riyadh, where it was observed that the mean correct score was 66.67% (13/20) [3]. To the best of our knowledge, there are limited studies on understanding the level of awareness among patients and their attendants affected by MSK and neurological disorders. Thus, it is imperative to create awareness among these groups of patients so as to enable lifestyle modifications, early detection and treatment in order to prevent further complications arising from osteoporotic fractures. Among the population affected by neurological disorders, there could be occurrence of pressure injuries, along with decreased range of motion and contractures of the hip and knee, thereby adding to the burden and strain of the patients and their caregivers [25]. Additionally, limited mobility and breaks in the skin integrity could contribute to development of pressure injuries and osteomyelitis at the fracture site. Manipulation of a fracture site could lead to severe hypertension arising from autonomic dysreflexia in SCI patients with neurological level of injury at or above T6 [25].

In our study, participants accompanying patients with neurological disorders and accompanying attendants (40.7%) were found to have poor knowledge about osteoporosis with an average score of 9.26 out of 20. In a study by Homann B et al., conducted among physically independent, community-dwelling individuals aged 60 and older with neurological disorders attending their Neurology OPD, it was found that 89% of stroke patients, 77% of those with Parkinson’s disease, 60% of dementia patients, and 57% of individuals with epilepsy had a notably high proportion of fallers [26]. Furthermore, the study revealed that 13.2% of patients with neurological disorders fell three or more times per year, compared to only 3.6% in the healthy control group [26]. Assistive devices are prescribed in patients with ambulation and mobility impairment due to neurological and musculoskeletal disorders, in order to decrease the risk of falling. These devices enable transfers and are used to manage balance and coordination disorders and muscle weakness, reducing energy expenditure, and ensuring safety, thereby facilitating activities of daily living [27].

A Physical Medicine and Rehabilitation physician helps prevent falls in patients with neurological disorders by assessing their fall risk and designing personalized exercise programs to improve strength, balance, and gait. They recommend assistive devices like canes or walkers and suggest home modifications for safety. The physician also educates patients and caregivers on fall prevention strategies including architectural modifications. Collaborating with a multidisciplinary team, they manage medication side effects and address cognitive or sensory impairments. Ongoing follow-up ensures that interventions are effective and adjusted as needed to improve safety and mobility [8,27].

Thus, it serves as an eye-opener for Physical Medicine and Rehabilitation physicians treating this cohort of patients with limited knowledge and awareness about osteoporosis and its risk factors. Osteoporosis awareness programs specially catering to such population, by means of conducting osteoporosis awareness campaigns in schools, using digital media to provide basic information regarding risk factors and treatment methods will improve the general understanding among the public. Additionally, a focused awareness program for this cohort of patients in a Physical Medicine and Rehabilitation setting will undoubtedly improve their knowledge and awareness of the disease, its risk factors, prevention and management.

The limitation faced in this study was that the evaluation was done in a cross-sectional pattern. Since the study mainly included patients and accompanying persons attending the OPD, it did not entirely represent the entire population of the community and was not homogeneous. Further studies could incorporate a questionnaire study tool assessment before and after hosting awareness campaigns.

## Conclusions

The assessment of osteoporosis knowledge and awareness in our study population using the OKAT-H scale highlights a significant gap in understanding. The findings indicate that many individuals with neurological and musculoskeletal disorders, as well as their caregivers or attendants, lack sufficient knowledge about the risks, prevention, and management of osteoporosis. This knowledge gap increases the risk of fractures and related complications. Therefore, it is essential to implement targeted educational programs for patients attending Physical Medicine and Rehabilitation departments for conditions such as spinal cord injury, stroke, and other neurological or musculoskeletal disorders.

To address this, targeted educational programs should be implemented in the OPD of Physical Medicine and Rehabilitation departments. These programs should focus on enhancing knowledge of osteoporosis, raising awareness of its risks and prevention, and educating individuals about fall prevention strategies, the use of mobility and orthotic aids, and necessary modifications to the home and workplace. The use of relevant tools in the local language, such as the OKAT-H scale, is vital for identifying these knowledge gaps and developing tailored interventions to meet the specific needs of this population.
